# Neuroprotection of retinal ganglion cells by the sigma-1 receptor agonist pridopidine in models of experimental glaucoma

**DOI:** 10.1038/s41598-021-01077-w

**Published:** 2021-11-09

**Authors:** Michal Geva, Noga Gershoni-Emek, Luana Naia, Philip Ly, Sandra Mota, Ana Cristina Rego, Michael R. Hayden, Leonard A. Levin

**Affiliations:** 1 Prilenia Therapeutics, Herzliya, Israel; 2 CNC-Center for Neuroscience and Cell Biology, University of Coimbra, Coimbra, Portugal; 3 The Centre for Molecular Medicine and Therapeutics, BC Children’s Hospital Research Institute, University of British Columbia, Vancouver, BC Canada; 4 FMUC-Faculty of Medicine, University of Coimbra, Coimbra, Portugal; 5 Department of Ophthalmology and Visual Sciences, McGill University, Montreal, Canada; 6 Department of Neurology and Neurosurgery, McGill University, Montreal, Canada; 7 Montreal Neurological Institute, McGill University, Montreal, Canada; 8 Present Address: Department of Neurobiology, Care Science and Society, Karolinska Institutet, Stockholm, Sweden

**Keywords:** Glaucoma, Translational research

## Abstract

Optic neuropathies such as glaucoma are characterized by retinal ganglion cell (RGC) degeneration and death. The sigma-1 receptor (S1R) is an attractive target for treating optic neuropathies as it is highly expressed in RGCs, and its absence causes retinal degeneration. Activation of the S1R exerts neuroprotective effects in models of retinal degeneration. Pridopidine is a highly selective and potent S1R agonist in clinical development. We show that pridopidine exerts neuroprotection of retinal ganglion cells in two different rat models of glaucoma. Pridopidine strongly binds melanin, which is highly expressed in the retina. This feature of pridopidine has implications to its ocular distribution, bioavailability, and effective dose. Mitochondria dysfunction is a key contributor to retinal ganglion cell degeneration. Pridopidine rescues mitochondrial function via activation of the S1R, providing support for the potential mechanism driving its neuroprotective effect in retinal ganglion cells.

## Introduction

Retinal ganglion cell (RGC) death is the final common pathway of all optic neuropathies as well as the most common irreversibly blinding disease in the world, glaucoma. One of the major risk factors of glaucoma is increased intraocular pressure (IOP). However, despite the availability of excellent treatments for lowering IOP, many patients will continue to lose their vision due to RGC death and degeneration of the optic nerve^1^. As a result, therapies aimed at the preservation of RGCs, their axons and dendrites are the focus of many years of research. These neuroprotective therapies have the potential for treating other diseases in which RGCs are damaged^2^.The pathogenic mechanisms leading to RGC death are common to several neurodegenerative disorders, and include endoplasmic reticulum (ER) and oxidative stress, mitochondrial dysfunction, disrupted signaling pathways and disturbances in Ca^2+^ homeostasis^1,3^.

The sigma-1 receptor (S1R) is a chaperone protein that is enriched at the ER-mitochondria associated membrane (MAM), where it plays a key role in the regulation of multiple cellular mechanisms, including enhancement of BDNF secretion, oxidative signaling, Ca^2+^ homeostasis, and mitochondrial function, among others^4–7^. Importantly, it is central to the cellular response to ER stress, and its activation mitigates ER stress and its downstream effects, directly affecting cell viability. In neurons, activation of the S1R improves neuronal connectivity, synaptic plasticity and elevates neurotransmitter expression^8,9^.

The S1R is an attractive target for the preservation of retinal ganglion cells (RGCs)^1^. It is highly expressed in the retinal ganglion cell layer, as well as in photoreceptors and the retinal pigment epithelium^10^. Mice lacking the S1R have late onset retinal degeneration, characterized primarily by RGC loss^11,12^. Electroretinographic (ERG) evidence of inner retinal dysfunction in S1R-knockout animals demonstrates not only morphological evidence of RGC death, such as increased numbers of terminal deoxynucleotidyl transferase dUTP nick end-labeling- (TUNEL-) positive cells, but also decreased numbers of cells, a decrease in the negative scotopic threshold response, and alterations in the optic nerve head^12^. The optic nerve head itself demonstrates axonal disruption and mitochondrial swelling. When the optic nerve is crushed in S1R-knockout mice, there is a higher rate of RGC loss compared to wild-type controls^13^. On the other hand, overexpression or activation of the S1R results in RGC neuroprotection^14^.

Oxidative stress and mitochondrial dysfunction occur in glaucoma patients, and can contribute both directly and indirectly to RGC degeneration^15^. For example, the level of superoxide anion increases within the cell body after RGC axonal injury, signaling the distal insult^16^. Elevated levels of reactive oxygen species (ROS) also trigger cell death in RGCs, which itself increases ROS levels, creating a deleterious feedback loop^15^. Indirectly, oxidative stress may contribute to RGC degeneration by driving an aberrant neuroinflammatory response or affecting vasculature, leading to reduced blood flow. For all these reasons, therapies targeting oxidative stress and mitochondrial dysfunction hold great promise in treating optic neuropathies.

Several studies have been carried out with different S1R agonists to determine whether S1R activation is neuroprotective in multiple models of retinal degeneration. In primary cultures of retinal ganglion cells exposed to glutamate and homocysteine, the S1R agonist (+)pentazocine (PTZ) decreases apoptosis^17^. In vivo, PTZ preserves retinal structure and protects retinal ganglion cells in diabetic Ins2^Akita/−^ mice, which typically have high rates of RGC loss^18^. Similarly, neuroprotection of RGCs is observed in response to treatment with the S1R ligands PRE-084 and (−)MR-22^19,20^.

Pridopidine is a highly potent and highly selective S1R agonist in clinical development for neurodegenerative diseases including Huntington disease (HD, NCT04556656) and amyotrophic lateral sclerosis (ALS, NCT04615923). The neuroprotective effects of pridopidine via S1R activation have been demonstrated in several in vitro and in vivo models of neurodegenerative diseases. Pridopidine upregulates secretion and transport of the brain-derived neurotrophic factor (BDNF) in models of HD, PD and ALS^9,21–23^. It restores dysregulated Ca^2+^ signaling and abnormal spine density as well as the homeostatic synaptic plasticity in HD neurons^8,24^. Pridopidine rescues the mutant huntingtin (mHtt)-induced decrease in mitochondrial function^25^ and cell death in both HD mouse neurons and human induced pluripotent stem cells (iPSCs)^26^. In the SOD1^G93A^ mouse model for ALS, pridopidine increases motor neuron survival, inhibits the disruption of neuromuscular junctions, restores the BDNF and mitochondrial transport deficiencies and restores synaptic activity, in a S1R-dependent manner^23^. The neuroprotective effects of pridopidine are also evident in models of Parkinson and Alzheimer diseases^22,27^ These effects are definitely S1R mediated because either pharmacological inhibition of the S1R or genetic deletion of the S1R gene completely abolish pridopidine’s protective effects.

In this study, we assessed the neuroprotective effects of pridopidine in two rat models of glaucoma, and the effect of pridopidine binding to melanin on dose selection. We further examined the direct effects of pridopidine on mitochondria function and ROS production as a potential mechanism for pridopidine’s neuroprotective effects for the treatment of retinal degeneration.

## Results

### Pridopidine protects retinal ganglion cells in the rat Morrison model of glaucoma

In order to test the efficacy of pridopidine for treating RGC degeneration and ultimate death, the well-established Morrison model for glaucoma was utilized. In this model, sclerosis of the aqueous veins via retrograde introduction of hypertonic saline increases IOP and usually does not recanalize.

Hypertonic saline injection (HSI) was performed on day 0 and day 7 into the episcleral veins of the right eye of pigmented Brown Norway rats in order to create a sustained increase of IOP. IOP and chronic ocular hypertension (OHT) induced neurodegeneration in the right eye (OD) similar to that in human patients with glaucoma, while the left eye served as control (OS).

**Figure 1:**
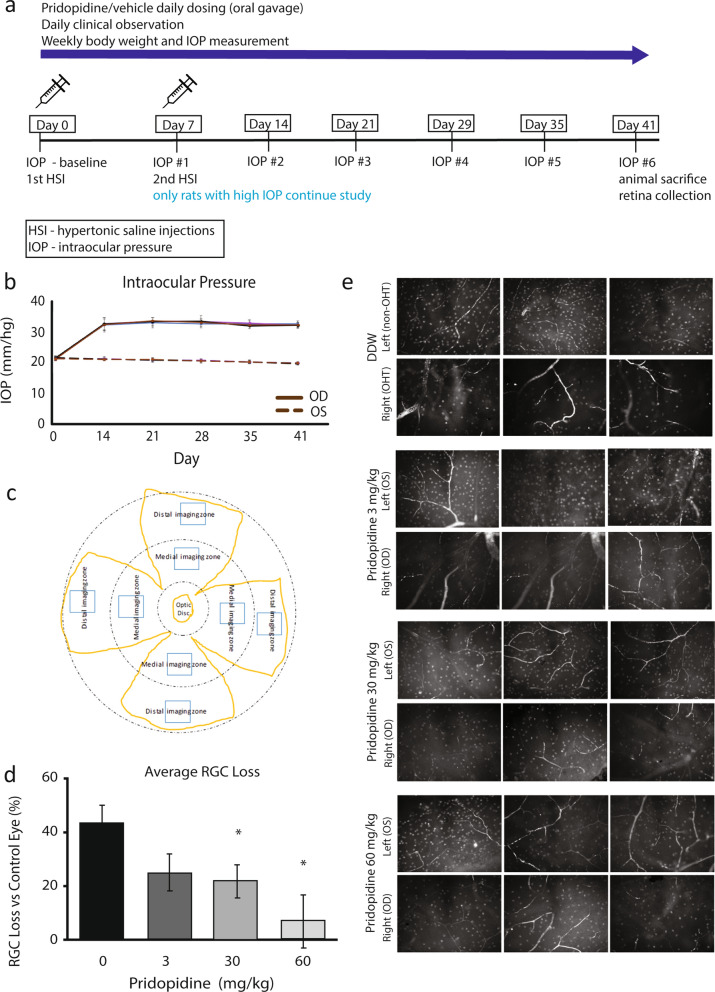
Pridopidine rescues RGCs at high doses in Morrison model for retinal neurodegeneration. (**a**) Diagram of study design. (**b**) Average IOP measured in OHT eye (OD) and non-OHT control (OS, dashed line) eye over 41 days of the study. (**c**) Diagram of regions of interest (ROI) collected from flat-mount retinas. Yellow outline—flat-mounted retina; Blue box—representative image location. (**d**) Average RGC loss per treatment group, calculated as the difference between the OD and the OS eye. (**e**) Representative images of retinas from treated rats stained for RGCs. Data is mean ± SEM. (n:DDW = 11, 3 mg/kg = 8, 30 mg/kg = 9, 60 mg/kg = 10; 4–8 images analyzed per eye). P < 0.05, One-way ANOVA.

Pridopidine at doses of 3, 30 and 60 mg/kg or control (double-distilled water, DDW) was administered daily by oral gavage, starting from day 1 after confirming IOP elevation until day 41. Over the course of the study, animals were clinically evaluated daily, and IOP and body weight measured weekly (Fig. 1a). There was no significant change in body weight (Supp Table 1; one-way ANOVA, P ≥ 0.05 for all groups at any given time point), and only mild corneal observations and retinal vessel enlargement, neither considered related to treatment, were found. In a control pilot study we confirmed that the highest dose of pridopidine (60 mg/kg) had no effect on IOP in normotensive Brown Norway rats (Supp Fig. 1).

Baseline IOP in both eyes ranged from 20.3 to 22.3 mmHg in all animals before administration of the first dose. By Day 14, 1 week after the second hypertonic saline injection (HSI), in the right eye (OD) the IOP increased by 11.1 ± 2.1 (mean ± SD) mmHg from baseline of all treatment groups, compared to − 0.3 ± 0.6 mmHg in control left eyes (OS) (Fig. 1b and Supp Table 2; Student’s t-test, p ≤ 0.0001). The elevation persisted until the end of the study (Fig. 1b).

Eligibility of animals for RGC analysis at the end of the study was determined by averaging the ΔIOP (ΔIOP = IOP_OD_-IOP_OS_) at four weekly timepoints (Days 21, 29, 35 and 41) (Supp Table 3). An animal was deemed eligible for further participation in the study if ΔIOP was greater than or equal to 6 mmHg. Mean ΔIOP in all animals ranged from 11.5 to 13.4 mmHg.

The number of RGCs was quantified using immunofluorescent staining for the RGC marker brain-specific homeobox/POU domain protein 3A (Brn-3a), followed by image analysis. RGCs were counted in one medial and one distal area in each retinal quadrant, up to eight regions per retina (Fig. 1c, Supp Table 4). The percent of RGC loss was calculated by comparing the number of RGC counts per retina in the OD eye to that in the OS eye. RGC loss (%) averages 43 ± 6% (mean ± SEM), 25 ± 7%, 21 ± 6%, 7 ± 9% in control, pridopidine 3 mg/kg, pridopidine 30 mg/kg, and pridopidine 60 mg/kg treated animals, respectively (Fig. 1d,e). Compared to vehicle control, pridopidine treatment results in RGC neuroprotection of 50% (p = 0.019) and 83% (p = 0.005) with the 30 and 60 mg/kg doses, respectively.

### Pridopidine binds strongly to melanin

The linear dose–response, with the highest dose being most efficacious, was surprising as pridopidine consistently demonstrates efficacy at low doses^8,9,26,27^.

The Morrison model was conducted in Brown Norway rats that have high melanin levels in the retina. We therefore hypothesized that the high concentration of melanin may affect pridopidine’s pharmacokinetics and availability due to potential pridopidine binding to melanin which may lower the drug’s free fraction available for S1R activation.

**Figure 2:**
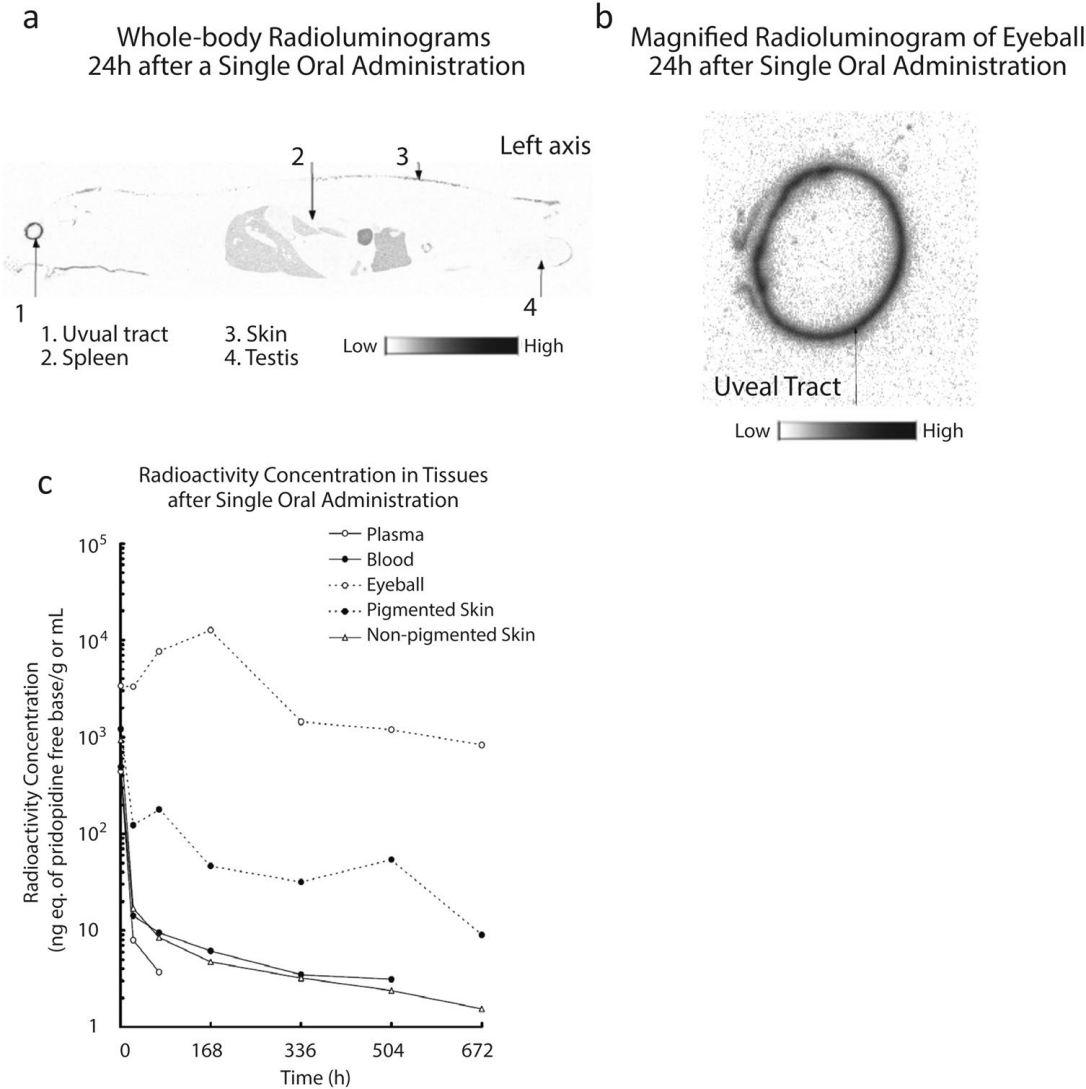
Pridopidine binds strongly to melanin. Food-deprived male rats were given 3 mg/kg of ^14^C-pridopidine free base, and radioactivity levels assayed at specific time points. (**a**) After 24 h, a whole-body radioluminogram was acquired, showing high levels in the highly pigmented uveal tract. (**b**) Magnified image of the uveal tract. (**c**) Relative levels of radioactivity in different tissues over time. Each data point is the values of one animal. A total of 7 animals was used.

To study this hypothesis, the retention of ^14^C-pridopidine in different tissues in the rat was evaluated. A single 3 mg/kg dose of ^14^C-pridopidine was administered to male Long Evans brown rats and radioactivity assessed over time in pigmented and non-pigmented tissues. At 24 h after administration, whole-body radioluminograms demonstrated a high level of radioactivity in melanin-containing uveal tissues (Fig. 2a), while low levels of radioactivity were detected in other tissues tested, including the skin and spleen. The magnified radioluminogram of the eye confirmed the high level of radioactivity in the uveal tract (Fig. 2b) and suggests that pridopidine has a strong binding affinity for melanin.

Quantification of radioactivity demonstrates increasing pridopidine binding in the eye after administration, reaching a maximal level at 168 h (1 week). Only at 672 h (4 weeks) after administration did the radioactivity concentration in the eye decrease, to 7% of its maximum. In contrast, other tissues (with the exception of pigmented skin) reached maximal pridopidine binding 1 h after administration. At 24 h after administration binding decreased to 3% or less of maximum, and at 72 h after administration decreased to 2% or less of the maximum (Fig. 2c).

These data confirm that pridopidine is retained in pigmented tissue, potentially binding strongly to melanin. We thus hypothesized that in the presence of melanin the fraction of free pridopidine available for binding the S1R is limited, and that the linear dose response observed in brown rats is due to pridopidine binding melanin. To test this hypothesis we assessed the neuroprotective effect of pridopidine on retinal ganglion cell loss in glaucomatous Wistar rats, which are albino and do not synthesize melanin.

### Pridopidine protects retinal ganglion axons in albino Wistar rats

**Figure 3:**
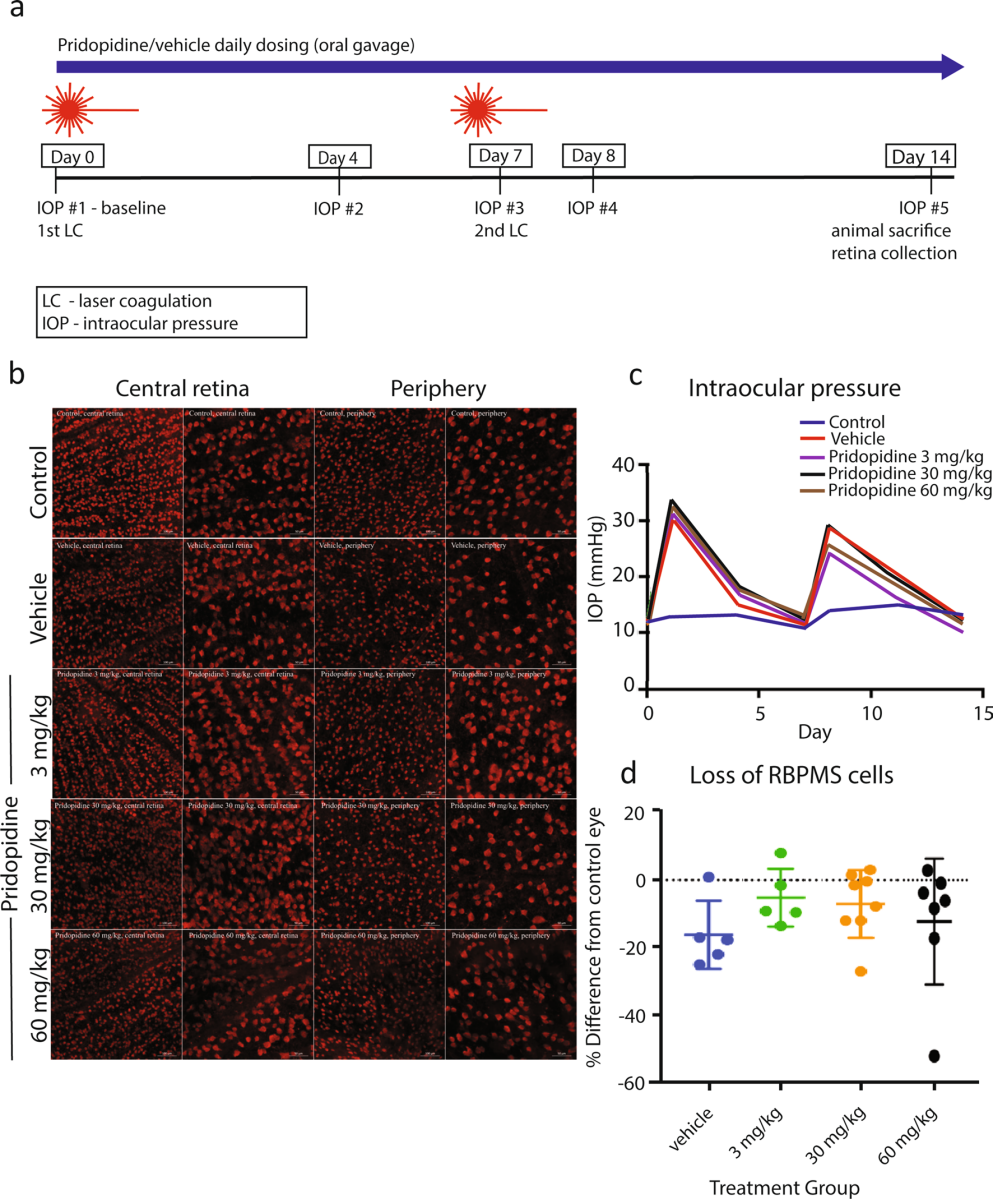
Pridopidine effect on RGC numbers in albino rats. (**a**) Diagram of study design. (**b**) Representative images of micrographs of RBPMS-positive RGCs from central part of retina (low and high magnifications) and peripheral part of retina (low and high magnifications, respectively) from different treatment groups: contralateral control, vehicle, pridopidine 3 mg/kg, pridopidine 30 mg/kg, pridopidine 60 mg/kg. Scale bar in low magnification images is 100 µm, and in high magnification images is 50 µm. (**c**) IOP in different treatment groups at a given study time point (day 0/baseline, day 1, day 4, day 7, day 8, day 11, day 14). (**d**) Loss of RBPMS-positive cells in different treatment groups as compared to contralateral control eyes after applying the 125/55 inclusion criterion. The data are presented as mean ± SD. One-way ANOVA followed by Dunn’s post hoc test. n = vehicle:6, 3 mg/kg: n = 5, 30 mg/kg: n = 9, 60 mg/kg:n = 7.

The laser coagulation (LC) model of experimental glaucoma was utilized in Wistar rats. Additional benefits of using this model are avoiding idiosyncratic features inherent to a single model and increasing the likelihood of future translatability to human glaucoma^28,29^. Both models utilize elevation of IOP, but to different degrees and with different time courses. Unilateral induction of IOP elevation was performed twice, at day 0 and day 7. Pridopidine at doses of 3, 30 and 60 mg/kg or vehicle control was administered by oral gavage daily, starting from day 1, after confirming IOP elevation (Fig. 3a). IOP was measured at baseline, and after laser photocoagulation on days 1, 4, 7, 8, and 14 (Fig. 3a). At the end of the study, RGC numbers were assessed by immunostaining (Fig. 3b).

Baseline IOP of both eyes ranged from 9.6 to 16 mmHg in all animals pre-treatment. Laser photocoagulation of the episcleral veins significantly increased IOP by 20.4 ± 7.4 mmHg from baseline in all treatment groups, compared to a change in control left eyes of 1.2 ± 2.3 mmHg from baseline (Fig. 3c and Table 1; Student’s t-test, p ≤ 0.0001). The peak elevation was within a day of laser photocoagulation and returned to baseline 7 days later. There was no difference in the IOP of lasered eyes based on the treatment administered (Fig. 3c; one-way ANOVA, followed by Dunn’s multiple comparisons test, p ≥ 0.26 for all groups). With respect to animal weight, there were no statistically significant differences between the groups at any time point analyzed (Supp Table 5; one-way ANOVA, P ≥ 0.25 for all groups at any given time point).

**Table 1:** Area under curve (AUC) of IOP in different treatment groups and in contralateral eyes of vehicle-treated animals (reference).

	Control	Vehicle	Pridopidine		
			3 mg/kg	30 mg/kg	60 mg/kg
Total area	185	272	255	289	295
Std. error	8.3	46	35	69	62

Because of the variability in IOP associated with the laser photocoagulation model, including high IOPs immediately after onset which could be associated with retinal ischemia or low IOP’s which could indicate failure to establish the model, we applied an inclusion criterion of initial IOP < 45 mmHg, and all animals with a cumulative IOP of > 125 mmHg were included, as long as no individual IOP measurement exceeded 55 mmHg at any time point. In addition, the IOP in the injected eye compared to the contralateral left eye had to be elevated by at least 6 mmHg after the second injection, and at no time greater than 55 mmHg.

The number of RGCs was quantified using immunofluorescent staining for RNA-binding protein with multiple splicing (RBPMS), an RGC-specific marker, followed by stereological analysis of the entire retina^30^ (Fig. 3c). In all groups, the number of RGCs in lasered eyes was significantly decreased compared to contralateral left control eyes (paired-samples t-test or Wilcoxon test; P ≤ 0.047 for all). In the vehicle-treated group, RBPMS-positive RGCs in the right eye decreased by 16.7% compared to the control left eye. There were no statistically different differences in total RGC loss identified between the groups (Table 2 and Fig. 3d; 1-way ANOVA followed by Dunnett’s post-hoc test). However, a trend appears for the 3 mg/kg and 30 mg/kg doses, at which cell numbers in the right eye decreased by 5.2% and 7.7% compared to the control left eyes, respectively. In the 60 mg/kg group RGCs decreased by 13.9% compared to control eye.

**Table 2:** The number of RGCs per retinal area of lasered eyes in different treatment groups with 125/55 exclusion criteria.

Eye	Vehicle	Pridopidine		
		3 mg/kg	30 mg/kg	60 mg/kg
**Contro**l				
Mean	1508	1404	1444	1417
STDEV	223	226	87	296
**Lasered**				
Mean	1280	1315	1336	1198
STDEV	234	112	178	295
**Difference**				
%	16.7	5.2	7.7	13.9

**Figure 4:**
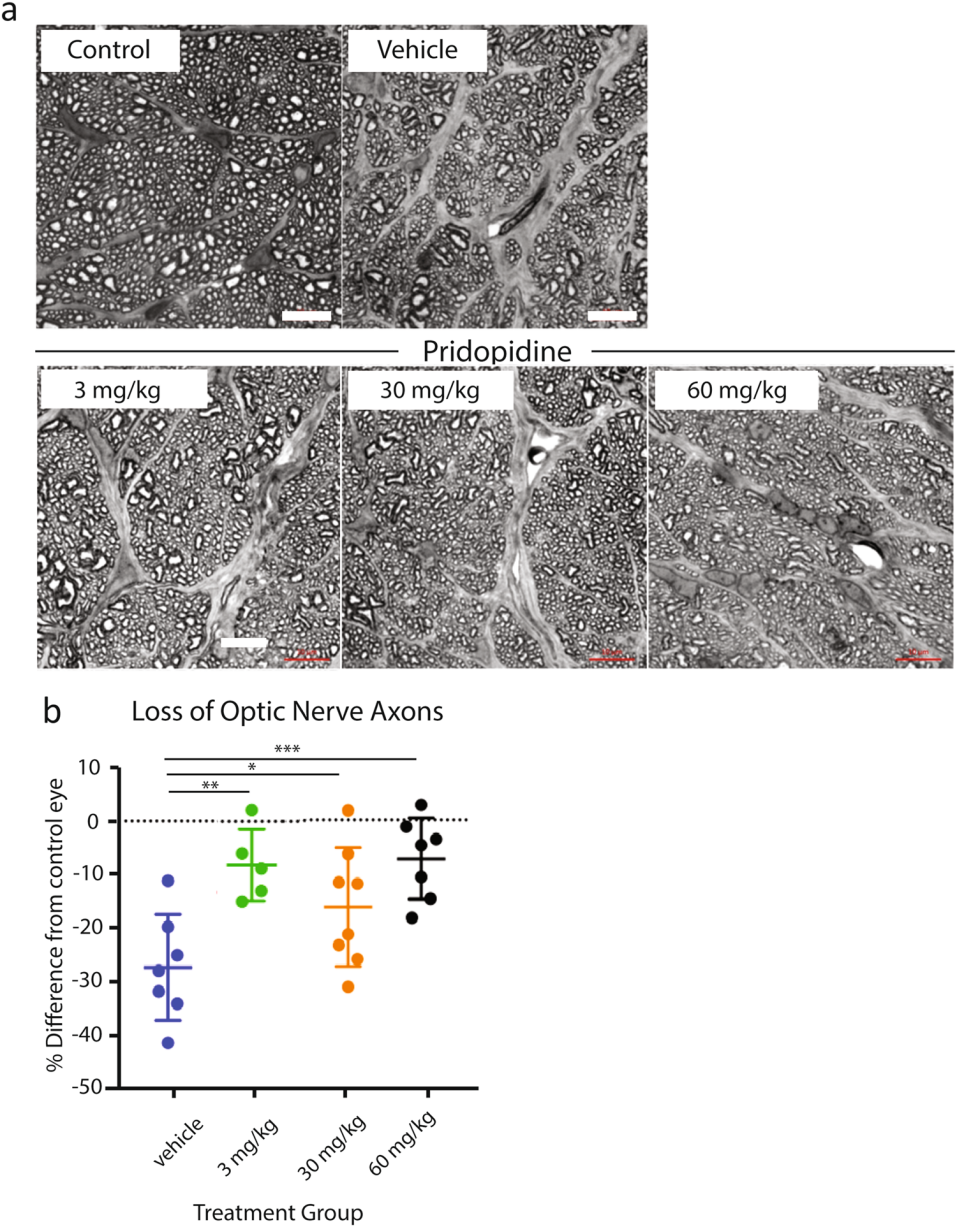
Pridopidine rescues optic nerve axons at a low dose in albino rats. (**a**) Representative micrographs of optic nerve sections acquired from semi-thin sections of optic nerves that were subjected to myelin enhancement staining. Note evidence of strongly increased gliosis in optic nerves from lasered eyes, as evident by regions stained in grey. Scale bar in all images is 10 µm. Images were taken from the following animals: control, vehicle, pridopidine 3 mg/kg, pridopidine 30 mg/kg, pridopidine 60 mg/kg (**b**). Scale bar in all images is 10 µm. (**c**) The loss of optic nerve axons in different treatment groups as compared to contralateral control eyes after the 125/55 inclusion criterion was applied. The data are presented as mean ± SD. 1-way ANOVA followed by Dunnett’s post-hoc test, *p < 0.05, **p < 0.01, ***p < 0.005. vehicle: n = 6, 3 mg/kg: n = 5, 30 mg/kg: n = 9, 60 mg/kg: n = 7.

Neuroprotective preservation of visual function in glaucoma and other optic neuropathies depends not only on preservation of cell bodies, but also on maintaining connectivity between the eye and the brain. Connectivity is preserved by protecting RGC axons^31–33^, i.e. *axoprotection*. To study this, the number of optic nerve axons per optic nerve area was compared between different treatment groups (Fig. 4a,b). The greatest loss of optic nerve axons was observed in vehicle animals (27.5% from control eye). Pridopidine 3 mg/kg, the lowest dose tested, demonstrates a significant protection of axon loss (only 8.6% loss of optic nerve axons vs. control eye, p = 0.006). Higher doses of pridopidine, 30 mg/kg and 60 mg/kg, similarly demonstrate a protective effect on optic nerve axons (15%, p = 0.04 and 7.24%, p = 0.002, respectively, 1-way ANOVA test followed by Dunnett’s post-hoc test) (Table 3, Fig. 4b). Compared to the vehicle group, pridopidine demonstrates axoprotection of optic nerves of 69, 45 and 74% at the 3, 30 and 60 mg/kg doses, respectively.

**Table 3:** Number of optic nerve axons in contralateral control eyes and in the laser-treated eyes after 125/55 inclusion criterion was applied.

Eye	Vehicle	Pridopidine		
		3 mg/kg	30 mg/kg	60 mg/kg
**Contro**l				
Mean	444	394	433	395
STDEV	58.3	28.5	62	36.3
**Lasered**				
Mean	323	358	363	365
STDEV	46	28	29	29
**Difference**				
%	16.7	8.6	15	7.6

### Pridopidine enhances mitochondrial functions and rescues mitochondrial membrane potential in NMDA-stressed neurons

Multiple mechanisms contribute to RGC death, including deprivation of neurotrophic factors, apoptotic cascade induction, immune system activation, calcium influx, mitochondrial dysfunction and signaling by cell-intrinsic reactive oxygen species (ROS)^34,35^. Chronic oxidative stress leads to mitochondrial dysfunction and the elevated generation of ROS, rendering RGCs especially susceptible to cell death^35^.

**Figure 5:**
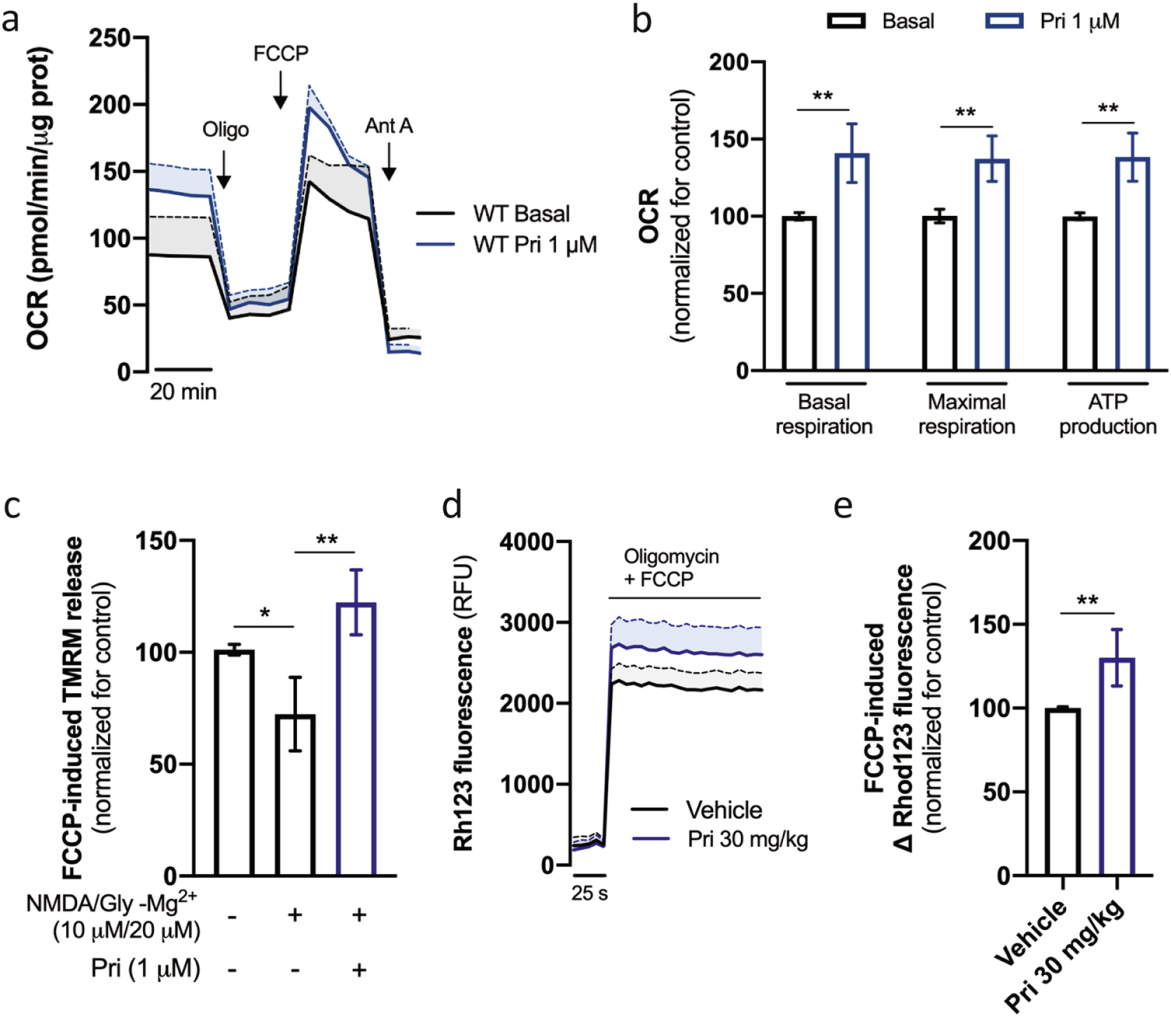
Pridopidine increases neuronal mitochondrial function. Striatal neurons were treated with 1 µM pridopidine for 24 h and OCR (oxygen consumption rate) was measured by Seahorse analyzer (**a**). Graph shows time-dependent changes in O_2_ levels (n = 6). (**b**) Quantification of OCR rate. (**c**) Striatal neurons were treated, when indicated, with 1 µM pridopidine for 24 h. 6 h before the experiment neurons were acutely stimulated, when indicated, with NMDA (10 µM) plus glycine (20 µM) for 15 min. TMRM fluorescence was recorded after complete mitochondrial membrane depolarization (n = 5). Mitochondrial membrane potential was measured in isolated striatal mitochondria using Rhodamine123 (**d**) and quantified (**e**). Graph shows time-dependent changes in fluorescence after adding oligomycin + FCCP (n = 6). Data are the mean ± SEM. All experiments were run in triplicates. *p < 0.05, **p < 0.01 by non-parametric Kruskal–Wallis test or Mann–Whitney test (in **e**).

Previous studies have shown a regulatory role of the S1R in mitochondrial function in retinal ganglion cells under stress^36–39^. The effects of pridopidine (1 μM) on mitochondrial function were therefore evaluated in mouse striatal neuronal cultures. Pridopidine significantly increases both basal and maximal mitochondrial respiration, as well as ATP production (p = 0.0082, p = 0.0061, and p = 0.0015 respectively, Mann Whitney test) (Fig. 5a,b).

A decrease in mitochondrial membrane potential (MMP) is indicative of reduced cellular health, and is often observed in neurodegenerative diseases such as glaucoma, Alzheimer disease, ALS and HD^40–43^.

Administration of pridopidine (1 μM) rescues the decrease in MMP induced by selective NMDA receptor activation using NMDA in the presence of glycine (p = 0.0072, one-way ANOVA followed by Dunn's multiple comparisons test) (Fig. 5c). The increase in MMP by pridopidine is further confirmed ex vivo by evaluating MMP levels in isolated mitochondria from pridopidine-treated mice (30 mg/kg daily for 45 days) (p = 0.0013, Mann Whitney test) (Fig. 5d,e).

### Pridopidine rescues H_2_O_2_-induced mitochondrial dysfunction by a S1R-dependent mechanism

We further investigated the effects of pridopidine on mitochondrial function and ROS production in human lymphoblasts. Hydrogen peroxide (H_2_O_2_) is a potent oxidative stressor, which induces ROS generation, leading to a decrease in MMP and cell death.

**Figure 6:**
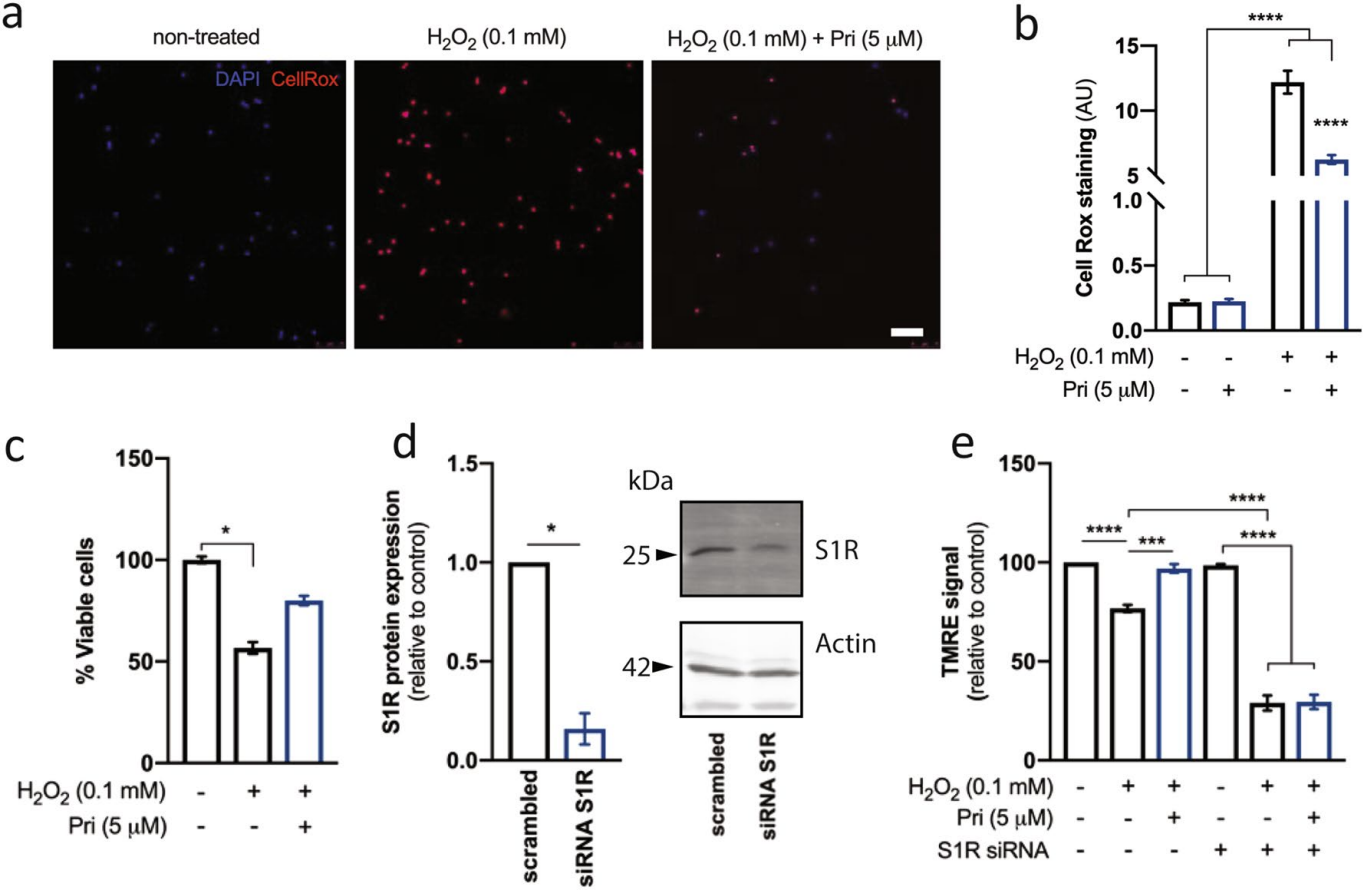
Pridopidine prevents H_2_O_2_-induced mitochondrial dysfunction and cell death in lymphoblasts through S1R. Human lymphoblasts were incubated with pridopidine (5 µM, 24 h) followed by H_2_O_2_ (0.1 mM, 6 h). ROS levels were quantified by co-staining with CellRox and DAPI. (**a**) Representative image and (**b**) quantification. Two-way ANOVA indicate an effect of H_2_O_2_ [F(1,12) = 373.8; p < 0.0001] and pridopidine [F(1,12) = 40.61; p < 0.0001] treatment. (**c**) Cell viability was determined by MTS assay. Lymphoblasts were subjected to S1R knockdown, confirmed by western blotting (**d**), and mitochondrial membrane potential was evaluated using TMRE (**e**). Data is the mean ± SEM. *p < 0.05, ***p < 0.001, ****p < 0.0001 by two-way ANOVA with a Tukey’s multiple comparisons in (**b**), by non-parametric Mann–Whitney in (**d**), or Kruskal–Wallis test in (**c**) and (**e**). Full-length blots are presented in Supplementary Fig. 2.

Human lymphoblasts incubated with hydrogen peroxide (0.1 mM) for six hours demonstrate a high degree of CellRox staining, indicative of ROS production. This effect is significantly ameliorated by pretreatment with 5 μM pridopidine for 24 h prior to H_2_O_2_ administration (p < 0.0001, 1-way ANOVA followed by Dunn's multiple comparisons test) (Fig. 6a,b). Furthermore, this effect on ROS production was mirrored by a trend towards reduction in cell death (Fig. 6c). Lymphoblasts treated with hydrogen peroxide demonstrate a decrease of approximately 40% in cell viability at 6 h. Pridopidine treatment (5 μM) shows a non-significant increase in cell viability up to 80 ± 4% of control (p = 0.36, one-way ANOVA followed by Dunn’s multiple comparisons test).

In order to assess whether these effects of pridopidine are mediated by the S1R, the S1R was genetically silenced with siRNA, achieving ~ 83% reduction in S1R protein levels in human lymphoblasts (Fig. 6d). Mitochondrial function was then assessed with the MMP-dependent probe TMRE (tetramethylrhodamine ethyl ester). In S1R-expressing cells, H_2_O_2_ reduces MMP by ~ 25%. Pridopidine treatment rescues this effect (p = 0.0002, one-way ANOVA followed by Dunn's multiple comparisons test). However, cells in which S1R was genetically silenced show higher sensitivity to H_2_O_2_, demonstrating a decrease of ~ 75% in MMP (p < 0.0001, one-way ANOVA followed by Dunn's multiple comparisons test). The effect of pridopidine is completely abolished in these cells, as pridopidine is unable to rescue MMP in the absence of S1R (Fig. 6e). Thus, the effect of pridopidine on MMP is S1R-mediated.

## Discussion

Validation of the S1R as a therapeutic target for the treatment of retinal degeneration come from several in-vitro and in-vivo studies, showing that activation of the S1R is neuroprotective.

In vitro, the S1R agonist SKF-10,047 attenuated cell death in the mouse cone photoreceptor cell line 661 W, and the high affinity S1R ligand (+) pentazocine (PTZ) had neuroprotective effects in primary rat RGC cultures^44^.

In vivo models include genetic models i.e., the rd10 mice Ins2Akita/+, a mouse model of diabetic retinopathy as well as induced models i.e. light-induced, in which photoreceptor degeneration is induced by excessive light exposure.

Mice carrying the retinal degeneration rd10 allele have a severe form of retinal degeneration, demonstrating loss of both rod and cone photoreceptor cells (PRs), within the first 6 weeks of life. Rd10 mice display impairments in ERG, abnormal retinal structure, and PR loss as well as gliosis and microglial activation. Treatment with PTZ improves ERG, decreases loss of retinal structure, and decreases gliosis and microglial activation compared to untreated animals^45^.

The Ins2^Akita/+^ diabetic mouse model of diabetic retinopathy demonstrates a retinal vascular phenotype in which retinal cell death and aberrant retinal architecture are apparent. PTZ preserves retinal architecture, and significantly decreases ganglion cell death in this model^46^.

In the light-induced photoreceptor death model SA4503 decreases retinal thinning and cell death and reduces mitochondrial damage associated with caspase activation^47^. Taken together, these results suggest that S1R activation has therapeutic potential to treat various eye diseases. However, not all S1R agonists show similar beneficial effects in all experimental models. Neither S1R agonists SA4503 nor PRE084 provide neuroprotection in the rd10 model^48^. Importantly, many of the S1R agonists are non-selective drugs, with high affinity for other receptors. For example, PTZ demonstrates a higher binding affinity to the Kappa opioid receptor (Ki = 2.2 nM)^49^ than to the S1R (Ki = 41 nM)^50^, and SA4503 demonstrates a higher affinity for empopamil binding protein (EBP, Ki = 1.72 nM) than for the S1R (Ki = 17.4 nM)^51^.

Pridopidine is a highly selective S1R agonist. The binding affinity of pridopidine to the S1R (Ki = 57 nM), is higher than its affinity for the dopamine D3 receptor (Ki = 1.6 3 μM) and the dopamine D2 receptor (Ki = 29. 5 μM)^52^.

Selectivity for the S1R is of high importance. Non-selective binding to additional targets may cause unwanted side effects^53^. Furthermore, low selectivity for the S1R vs. the sigma 2 receptor (S2R) and activation of the S2R, antagonizes the beneficial effects of S1R^53^.

We studied the potential therapeutic effects of pridopidine in two different experimental glaucoma models in which retinal degeneration is induced by elevated IOP (Figs. 1, 3, 4). Pridopidine demonstrates a neuroprotective effect on RGCs in both models. Interestingly, pridopidine’s protective effect is not mediated by lowering IOP, suggesting therapeutic potential for optic neuropathies beyond glaucoma.

The mitochondrial membrane potential (MMP) is critical for maintaining mitochondrial health and function. Impaired MMP is common to numerous degenerative neuroretinal diseases, including retinitis pigmentosa and age-related macular degeneration, and most relevantly, Leber hereditary optic neuropathy (LHON)^54,55^. Furthermore, impaired MMP leads to apoptosis and RGC death^56^. Thus, MMP rescue is an attractive therapeutic target for retinal neuropathies^56^. Indeed, idebenone, a drug approved in Europe for the treatment of LHON, rescues impaired MMP and increases RGC survival^57,58^.

The protective effects of pridopidine are potentially mediated by rescue of MMP and improvement of overall mitochondrial functions in the presence of an oxidative stress injury (Fig. 5), mediated by S1R (Fig. 6).

For decades it has been difficult to develop drugs that are neuroprotective both in in vitro and in animal models, and which can also translate into efficacy in human studies of disease^59^. Furthermore, an in vitro effect does not necessarily translate into an in vivo effect^48^. The data garnered from the two glaucoma models used in this study, coupled with the mechanistic and pharmacological studies, together provide a rationale for continuing the investigation of pridopidine in early-phase clinical trials in glaucoma, and potentially other optic neuropathies. The finding that two different animal models of glaucoma both show neuroprotection against RGC loss, albeit with some differences, supports the notion that the protective effect of pridopidine on RGCs is not idiosyncratic to the specific methodology used in each study. The optimal pridopidine dose for neuroprotection was different in the two models. In the Morrison hypertonic saline injection model in brown rats (Fig. 1), maximal RGC survival was seen at the high doses (30 and 60 mg/kg), while in the laser photocoagulation model in albino Wistar rats, pridopidine at a low dose (3 mg/kg) was neuroprotective (Fig. 4).

There are several potential factors that may contribute to the observed differences in pridopidine’s dose response in the two models.

One potential explanation for the difference in pridopidine’s dose response between the two animal models may be attributed to differences in the amount of free drug in the retina. The laser photocoagulation study was performed with Wistar rats, which are albino and have insignificant levels of melanin in the retina. The Morrison model study was performed with Brown Norway animals, which are pigmented. The greater amount of melanin in the latter would bind pridopidine and decrease its free fraction available to activate S1Rs. On the other hand, this binding could also serve as a depot and might affect dose-dependence in longer term studies.

Furthermore, the time course of the axonal injury in the two animal studies is different. In the Morrison model, animals were assessed over a period of 41 days, while in the laser coagulation model (LC) animals were assessed after 14 days. It is possible that the Morrison model is evaluating the long-term effects of pridopidine on processes such as axonal maintenance, and its short-term effects on processes such as ROS production in the LC model.

In addition, the two models have intrinsic differences including the dynamics of IOP elevation and the type of damage inflicted by the model. Although great care was taken to analyze data only from animals that had appropriate IOP elevation for each model used (not too low and thus insufficient to cause RGC death, and not high enough to cause ischemia), the models have intrinsic differences that are relevant to human glaucoma. In the laser photocoagulation model, there is an acute rise in IOP, which is sustained for several days and then returns to baseline. Therefore, in this model a second surgical procedure is needed to achieve sufficient IOP elevation and cause stable and reliable RGC death. This model is therefore more relevant to patients with variable IOPs.

In contrast, the IOP is better sustained over time in the Morrison hypertonic saline model. In this model, a single hypertonic injection results in a sustained increase in IOP, and sustained and reliable RGC death is achieved 40 days post injection and may be more representative of patients with less variable IOP. The differences in time course of IOP in the two models could impact the neuroprotective effect due to differences in the dynamics of axonal injury, signaling by reactive oxygen species, activation and half-life of transcription factors, and effects of S1R occupancy and downstream effects.

The answers to these hypotheses could be studied in future mechanistic and pharmacokinetic experiments, but in the end, a phase 2 dose-ranging trial would best determine the optimal clinical dose of pridopidine for assessing glaucoma neuroprotection in a patient population with specific inclusion and exclusion criteria.

The mechanism of pridopidine neuroprotection includes protection of mitochondrial function, based on the experiments demonstrating a S1R-mediated stabilization of mitochondrial membrane potential in response to oxidative stress. Our previous studies demonstrate that superoxide anion, or potentially an interconvertible reactive oxygen species, is a signaling molecule for RGC death after axonal injury^60,61^. These studies used in vivo imaging to demonstrate a rise in superoxide anion after optic nerve injury. We previously demonstrated that drugs which decrease superoxide levels, or more downstream, drugs that reduce disulfide bonds^62–64^, are neuroprotective for both RGC somas and axons in optic nerve models. Therefore, our findings of an S1R-dependent mechanism of action by which pridopidine stabilizes mitochondria in the presence of oxidative stress is relevant to axonal injury.

It is becoming increasingly clear that mitochondria play crucial roles in the cell, beyond providing the necessary bioenergetics for its proper function. Mitochondria are central regulators of cell survival and ROS production, and also important for intracellular signaling and gene expression^65,66^. At the MAM, ER and mitochondria create contact sites that govern cellular functions inherent to cellular survival. Due to its location at the MAM, the S1R acts as a bridge between ER function and mitochondria and pridopidine enhances these contacts in physiological/wild-type conditions^25^. The S1R participates in the maintenance of mitochondrial integrity by regulating Ca^2+^ flux between the ER and the mitochondria, governs cell survival by modulating the cellular response to stress, and is essential for regulating autophagy^65,67^. Accordingly, the MAM hosts a nanodomain of oxidative species which is induced by cytosolic Ca^2+^ spikes^68^ The S1R is critical to the regulation of ROS production, as its genetic deletion or pharmacological inhibition results in increased ROS levels. On the other hand, S1R overexpression or activation induces the Nrf2-ARE gene pathway that decreases ROS levels^25,69^.

Future studies aimed at elucidating the distinct sites and mechanistic pathways for pridopidine neuroprotection via S1R activation may include in vivo imaging of mitochondrial membrane potential, assessment of mitochondrial complex activity and Ca^2+^ flux in vitro and in vivo.

Brain-derived neurotrophic factor (BDNF) plays a significant role in retinal ganglion cell neuroprotection^70–73^ and increasing BDNF signaling in the eye is a promising therapeutic avenue. However, clinical development is impeded by challenges to effectively deliver BDNF to the eye. A drug that can effectively increase BDNF in the retina is a highly attractive candidate for the treatment of optic neuropathies^74,75^. Pridopidine upregulates BDNF secretion and transport via activation of the S1R in several models of neurodegeneration including models of HD, PD and ALS^9,21–23^.The effect of pridopidine on BDNF may potentially contribute to its observed protection of RGCs.

The S1R is a highly promising target for treating retinal neurodegeneration, with a growing body of evidence supporting it as a target for treating retinal cell dysfunction. A recent study showed that not all S1R agonists are equal. While (+) pentazocine (PTZ) has beneficial effects both in vitro and in vivo, other S1R activators SA4503 and PRE084 had only beneficial in vitro effects that did not translate to in vivo rescue of RGCs in the rd10 mouse model of retinopathy^48^. These data in two different in vivo models further highlight the translational potential of pridopidine for therapeutic purposes.

In summary, we show that pridopidine exerts neuroprotective effects in RGCs in animal models of glaucoma. These effects are mediated by the S1R and are likely related to mitochondrial function and regulation of ROS levels. Taken together with the established safety and tolerability profile of pridopidine in clinical studies, our study highlights the translatability of pridopidine from preclinical models and supports advancement of the clinical development of pridopidine for glaucoma and possibly other optic neuropathies.

## Methods

### Animals

Animals in all experiments were treated in accordance with local ethical guidelines (detailed per experiment below). Animals were housed under controlled temperature conditions (20–26 °C) with a 12-h light/dark cycle. Food and water were available ad libitum. Animals were acclimated for at least 5 days before experimentation. Animal studies are reported in accordance with ARRIVE guidelines (https://arriveguidelines.org).

### Experimental glaucoma by hypertonic saline injection into aqueous veins (Morrison model)

Animals were treated in accordance with the ARVO Statement for the Use of Animals in Ophthalmic and Vision Research, the United States Department of Agriculture Animal Welfare regulations, and the Office for Laboratory Animal Welfare (OLAW) Public Health Service Policy on Humane Care and Use of Laboratory Animals.

#### IOP elevation

Rats were sedated with ketamine and xylazine (40–80 and 5–10 mg/kg, respectively), kept warm and eyes moist to avoid desiccation during the procedure. Eye surface was treated with erythromycin (0.5%). On Days 0 and 7, hypertonic saline solution (250 μL, NaCl, 1.8–2.0 M) was injected into the limbal vascular plexus via different episcleral veins in the OHT eye.

Corneas were anaesthetized with 0.5% proparacaine HCl ophthalmic solution and IOP measured using a Tono-Pen Vet. Ten repeated readings were averaged from each eye.

#### Assessment of RGC survival

Eyes were immediately enucleated following CO_2_ euthanasia and fixed in paraformaldehyde (PFA, 4%) at 4 °C for 24 h, then dissected in PBS. Retinas were permeabilized in PBS + 0.5% Triton X-100 for 15 min, frozen at − 80 °C for 15 min, then rinsed in PBS-0.5% Triton. Samples were incubated overnight at 4 °C with anti-Brn-3a primary antibody (Brn-3a 14A6: #sc-8429) in blocking solution (PBS, 2% normal donkey serum, 2% Triton X-100). Retinas were washed 3× in PBS and incubated with fluorescent secondary antibodies (anti-mouse IgG (H + L), Alexa Fluor 594, #A21203) in blocking buffer, then washed 3 times. Four radial cuts were made in the retina and it was flat-mounted. Two regions of interest (medial and distal) were acquired in each quadrant with fluorescent microscopy, and RGCs counted using ImageJ.

### Experimental glaucoma by laser photocoagulation of episcleral veins (LC model)

Male 5 months old Wistar (RjHan:WI) rats were treated in accordance with the ARVO Statement for the Use of Animals in Ophthalmic and Vision Research and the EC Directive 86/609/EEC for animal experiments, using protocols approved and monitored by the Animal Experiment Board of Finland (Experimentica Ltd. animal license number ESAVI-4139-2017).

#### IOP elevation

Experimental IOP increase was induced unilaterally by 532 nm diode laser (Oculight® TX; Iridex) photocoagulation of episcleral veins, as previously described^30^. IOP was measured with a TonoLab tonometer. All lasered eyes had IOP of 18 mmHg or higher as assessed on day 1.

#### Animal sacrifice and tissue collection

Compounds were administered daily by oral gavage. On day 14 rats were sacrificed by transcardial perfusion, first with 0.9% NaCl solution, then with 4% PFA in 0.1 M phosphate buffer, pH 7.4. Eyes and optic nerves were collected for immunohistochemical analysis.

#### RGC and axon assessment

Retinal flat-mounts were immunostained with rabbit anti-RBPMS (PhosphoSolutions) and DAPI nuclear stain. Total number of RBPMS-positive cells was estimated using stereology as previously described^30^.

Optic nerves were postfixed in 4% PFA (in 0.1 M phosphate buffer, pH 7.4), placed in 1% osmium, dehydrated in ascending alcohol concentrations, and placed in 1% uranyl acetate in 100% ethanol for 1 h^76,77^. Optic nerves were embedded in epoxy resin mixture at 60 °C for 48 h and 1 µm sections prepared. Axon number was estimated using stereology, as previously described^77^.

### Data analysis

Data was analyzed using GraphPad Prism 7 for Mac OS X (version 7.0a). Data were analyzed using one-way ANOVA with Dunnett’s test for post hoc multiple comparison testing. Statistical significance was achieved at *p* < 0.05.

### Melanin binding

Long Evans male rats were fasted from ~ 16 h before to 4 h after administration. All procedures were carried out under the approval of the Animal Ethics Committee of ADME/TOX Research Institute, Daiichi Pure Chemicals Co., Ltd.

#### Radioactivity measurements

Purified ^14^C-labeled pridopidine hydrochloride was used, and radiochemical purity determined periodically. HPLC analysis of radiochemical purity was performed before the first use and after the final use. To confirm the elution site of pridopidine, unlabeled pridopidine HCl was dissolved in distilled water/acetonitrile (9:1, vol/vol).

^14^C-pridopidine was dissolved in distilled water/acetonitrile (9:1, vol/vol), and analyzed by HPLC on an Ace C18-100 5 μm, 4.6 mm I.D. × 250 mm L column at ambient temperature. The mobile phase consisted of A) 20 mmol/L KH_2_PO_4_ and 5 mmol/L K_2_HPO_4_ and B) acetonitrile. Approximately 500,000 dpm radioactivity was injected, for a run time of 33 min at 1.0 mL/min flow rate and 215 nM UV detection.

Radioactivity in the HPLC eluate was monitored using RAD (radioactivity detector, Perkin Elmer). Scintillator Flo-Scint II was delivered to the HPLC eluate at threefold flow rate of the mobile phase. Radioactivity (cpm) was counted with 6 s integration using RAD. Detection limit of radioactivity was defined as 2 × background value. Radiochemical purity: [purity (%) = (S/T) * 100]; S: counts at elution site, T: total counts over run time. Column recovery: [column recovery (%) = (R/I) * 100]; R: radioactivity in the eluate over run time, I: injected radioactivity.

#### Radioactivity measurement in tissues

^14^C-pridopidine was dissolved in water for a solution of 3 mg free base/6.475 MBq/mL, aliquoted and stored at 4 °C. Dosing solution (3 mg free base/1 mL/kg) was administered once to fasted rats by oral gavage.

Animals were sacrificed by exsanguination from the abdominal vena cava under ether anesthesia. Tissues were excised, and radioactivity concentrations determined. Blood was collected from the abdominal vena cava. Plasma was separated from the remaining blood. Tissues were collected and solubilized with 2 mL of Soluene-350 with heating. Samples were mixed with 10 mL of the scintillator Hionic-Fluor (Perkin Elmer), and radioactivity measured using LSC (Liquid Scintillation Counter, Perkin Elmer). Radioactivity in blood cells was calculated using the radioactivity concentrations in blood and plasma.

Radioactivity (dpm) was counted using LSC for 2 min after scintillator addition. Counting efficiency was corrected by the external standard source method. Detection limit was defined as 2 × background value.

#### Background radioactivity

The following samples underwent radioactivity assay: dosing solution, the solvent used for dilution; column recovery of solution, HPLC mobile phase; biological samples, samples from untreated animals, and blood from an untreated animal (to represent untreat tissues).

Radioactivity concentration was calculated using the data processing system (ADMEDAMS Ver.2.02). Radioactivity concentration (ng eq. of pridopidine free base/g or mL) = (D − B)/(F × S); D: Radioactivity in assay sample (dpm) B: Background value (dpm), F: Specific radioactivity of dosing solution (dpm/ng), S: Amount of assay sample (g or mL). Radioactivity counts below the detection limit are expressed as ND (Not detected). Result is expressed as mean value ± standard deviation (SD) of group animals (three animals). Ratio of tissue radioactivity at each time point to the maximum tissue concentration (Ct/Cmax ratio), was calculated from mean values obtained at each time point.

#### Whole-eye radioactivity measurement

Eyes were dried in a filter cup at 40 °C for at least 24 h and combusted. Generated ^14^CO_2_ was absorbed in 6 mL of CO_2_ absorbent Carbo-Sorb E and 12 mL of scintillator Permafluor E+ was added. Radioactivity in the mixture was measured using LSC. Measurements from the combustion method were corrected with recovery, calculated using the equation below using 0.2 mL of ^14^C standard sample (Spec-Chec ^14^C) for sample oxidizer. Recovery was calculated as radioactivity recovered after standard sample combustion divided by the radioactivity of the standard sample subjected to combustion (dpm).

#### Whole-body autoradiography

After a single ^14^C-pridopidine administration, animal was sacrificed with ether overdose, and whole-body autoradiograms prepared. Hair was clipped, the nasal cavity and anus filled with 4% carboxymethyl cellulose sodium (CMC-Na), and the carcass frozen in a dry ice-acetone mixture. After removal of extremities, carcass was embedded in 4% CMC-Na, frozen again in dry ice-acetone, sectioned in a cryomicrotome (30 μm) and collected onto adhesive tape. Sections were lyophilized for 3 days, then covered with a protective membrane (4 μm, Diafoil) and placed in contact with an imaging plate for one day exposure in a sealed lead box. Radioactivity was analyzed using BAS2500.

### Tissue culture

#### Lymphoblasts

Lymphoblasts (Corriel Institute, GM02174) were grown in RPMI medium containing 10% FBS, 2 mM l-glutamax, and 100 U/mL penicillin–streptomycin and passaged every 5–6 days. Pridopidine was added to the media for 24 h, followed, when indicated, by 6 h of H_2_O_2_ treatment (0.1 mM). S1R knockdown was achieved using S1R-directed siRNA duplexes (Origene, cat no: SR426072). Transfection with Polyplus JETPRIME transfection protocol was performed 48 h before experiments. S1R knockdown was assessed by immunoblot.

#### Primary neuronal cultures

Primary striatal cultures were generated from FVB/N mice. All mouse protocols were approved by the Faculty of Medicine, University of Coimbra (ORBEA_189_2018/11042018) and carried out in accordance with the guidelines of the Institutional Animal Care and Use of Committee and the European Community directive (2010/63/EU).

Striatal neurons were isolated as previously described^78^ from embryos at gestational day 16–17. Striata were dissected, dissociated with trypsin (0.5 mg/mlL, then neutralized with trypsin inhibitor solution (1 mg/mL), both in ice-cold HBSS with 0.3% fatty acid free BSA. Neurons were cultured in Neurobasal medium with 2% B27, 1 mM glutamine, 20 μg/mL gentamicin and plated at a density of 130 × 10^3^ cells/cm^2^ in poly-d-lysine (0.1 mg/mL). Glial inhibitor 5-fluoro-2′-deoxyuridine (5 μM) was added at DIV3. At 12DIV, N-methyl-d-aspartate (NMDA, 10 μM) and co-agonist glycine (20 μM) were added to the culture for 15 min followed by 6 h recovery to gate extrasynaptic glutamatergic NMDA receptors^79^. Pridopidine was added for 24 h.

### ROS staining

Lymphoblasts were attached to poly-d-lysine-coated plates and treated with 5 µM CellRox red reagent in complete medium for 30 min. All samples were washed with PBS and imaged using identical exposure settings. Eight random fields were sampled, and fluorescence intensity normalized to the DAPI signal.

### Mitochondrial membrane potential measurement in vitro

Lymphoblasts were incubated with TMRE (25 nM) in cell media for 15 min at 37 °C, centrifuged and re-suspended in PBS with 1% FBS for FACS analysis using a PE filter (545 nm). Neurons were incubated with 150 nM TMRM (tetramethylrhodamine methyl ester) in Na^+^ medium (in mM: 140 NaCl, 5 KCl, 1 CaCl_2_, 1 MgCl_2_, 10 glucose, 10 HEPES, pH 7.4) for 30 min at 37 °C. FCCP (2.5 μM) with oligomycin (2.5 μg/mL) was used to depolarize mitochondria and release TMRM. Fluorescence (503 nm excitation, 525 nm emission) was recorded using a microplate reader.

### Cell viability

Lymphoblasts were incubated with MTS reagent (Promega) in culture media (1:10) for 4 h at 37 °C, then centrifuged at 145×*g* for 5 min. Absorbance was quantified in media at 490 nm.

### Mitochondria isolation and membrane potential measurement ex vivo

Mitochondria were isolated from striata using discontinuous Percoll density gradient centrifugation as previously described^80^ and resuspended in ice-cold washing buffer (in mM: 250 sucrose, 5 HEPES–KOH, 0.1 EGTA, pH 7.2). For MMP measurement, 10 μg mitochondria were incubated in reaction buffer (in mM: 100 sucrose, 100 KCl, 2 KH_2_PO_4_, 5 HEPES, 0.01 EGTA, 3 succinate, 3 glutamate, 0.1 ADP-K, pH 7.4) with 50 nM Rhodamine123. Basal fluorescence was measured every 30 s for 5 min (excitation: 503 nm; emission: 525 nm). A mixture of FCCP(2 μM)/oligomycin (2 μg/mL) was added to induce mitochondrial depolarization, and fluorescence measured.

## Supplementary Information

Supplementary Information.
