# Supplementary material for: Neuroprotection of retinal ganglion cells by the sigma-1 receptor agonist pridopidine in models of experimental glaucoma
Source: Sci Rep. 2021 Nov 9;11:21975. doi: 10.1038/s41598-021-01077-w (PMC8578336; doi:10.1038/s41598-021-01077-w)
Supplement: Supplementary file 1 — Supplementary Information. [file 41598_2021_1077_MOESM1_ESM.docx]

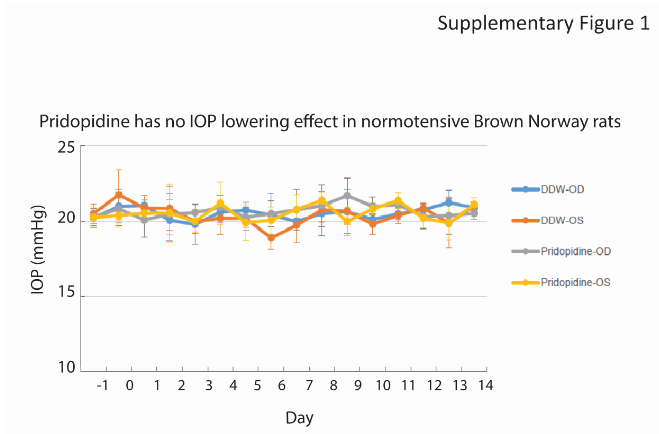


**Supplementary Figure 1**

**Pridopidine does not affect IOP in normotensive Brown Norway rats**

Brown Norway rats were treated with 60 mg/kg pridopidine or an equal volume of double distilled water (DDW) for 14 days (n=6/group). IOP was measured pre-dose (Day -1), and daily on days 0-13 in both eyes of awake animals. Ten continuous readings were taken from each eye with a Tono-Pen Vet, and the mean calculated. Data presented as mean ± SEM. OD – disease eye, with ocular hypertension (OHT); OS – control eye, non-OHT.

Supplementary Figure 2 (corresponds to Figure 6d)

**Supplementary Table 1: Summary of Body Weights - Morrison model**

|  | | | | | |  |  |  |  |
| --- | --- | --- | --- | --- | --- | --- | --- | --- | --- |
|  |  |  | Day | | | | | | |
|  | Group |  | 0 | 7 | 14 | 21 | 28 | 35 | 41 |
|  | DDW | Mean (g) | 243.35 | 248.5 | 246.48 | 247.23 | 257.93 | 249.18 | 250.05 |
|  |  | S.D. | 10.3 | 10.9 | 11.56 | 11.54 | 11.63 | 11.56 | 11.4 |
|  |  | N | 12 | 12 | 12 | 12 | 12 | 11 | 11 |
| Pridopidine | 3 mg/kg | Mean (g) | 244.58 | 250.96 | 248.35 | 249.97 | 250.34 | 251.39 | 251.83 |
|  |  | S.D. | 10.78 | 11.09 | 12.23 | 11 | 11.08 | 10.79 | 11.2 |
|  |  | N | 12 | 11 | 11 | 11 | 11 | 11 | 11 |
|  | 30 mg/kg | Mean (g) | 246.52 | 252.05 | 249.25 | 249.97 | 250.63 | 251.59 | 252.09 |
|  |  | S.D. | 10.93 | 11.1 | 11.48 | 11.8 | 11.68 | 11.29 | 11.42 |
|  |  | N | 12 | 12 | 12 | 12 | 12 | 12 | 12 |
|  | 60 mg/kg | Mean (g) | 245.84 | 251.66 | 249.71 | 250.75 | 252.73 | 253.68 | 254.1 |
|  |  | S.D. | 10.91 | 11.2 | 11.59 | 11.06 | 10.85 | 10.52 | 10.96 |
|  |  | N | 12 | 12 | 12 | 12 | 11 | 11 | 11 |

**Supplementary Table 2: Weekly assessment of IOP in test and control eyes - Morrison model**

|  |  | OD | | | OS | | |
| --- | --- | --- | --- | --- | --- | --- | --- |
| Treatment Group | Subject | IOP1 - Day 0 (mmHg) | IOP2 - Day 14 (mmHg) | ΔIOP (mmHg) | IOP1 - Day 0 (mmHg) | IOP2 - Day 14 (mmHg) | ΔIOP (mmHg) |
| DDW | 1001 | 22.4 | 30.6 | 8.2 | 21.9 | 20.7 | -1.2 |
|  | 1002 | 21.5 | 30.3 | 8.8 | 22 | 21.6 | -0.4 |
|  | 1003 | 22.2 | 29.4 | 7.2 | 21.5 | 20.7 | -0.8 |
|  | 1004 | 21 | 30.3 | 9.3 | 22.3 | 21.4 | -0.9 |
|  | 1005 | 20.3 | 30.6 | 10.3 | 21.2 | 21.7 | 0.5 |
|  | 1006 | 20 | 32.6 | 12.6 | 20.7 | 20.7 | 0 |
|  | 1007 | 21.7 | 35 | 13.3 | 21.8 | 21.7 | -0.1 |
|  | 1008 | 21.8 | 35.4 | 13.6 | 22 | 21.7 | -0.3 |
|  | 1009 | 21 | 29.6 | 8.6 | 20.5 | 20.8 | 0.3 |
|  | 1010 | 20.6 | 34.4 | 13.8 | 21.2 | 20.8 | -0.4 |
|  | 1011 | 22.7 | 33.3 | 10.6 | 21.8 | 21 | -0.8 |
|  | 1012 | 21.4 | 34.2 | 12.8 | 20.6 | 20.7 | 0.1 |
| 3 mg/kg | 2001 | 21.8 | 29.4 | 7.6 | 21.4 | 21.9 | 0.5 |
|  | 2002 | 20.8 | 29.7 | 8.9 | 20.6 | 20.8 | 0.2 |
|  | 2003 | 21.3 | 30.2 | 8.9 | 20.3 | 20.9 | 0.6 |
|  | 2004 | 20.8 | 30.4 | 9.6 | 21.3 | 20.8 | -0.5 |
|  | 2005 | 21.7 | N/A |  | 21.5 | N/A |  |
|  | 2006 | 20.8 | 32.5 | 11.7 | 22.2 | 20.9 | -1.3 |
|  | 2007 | 21.4 | 34.1 | 12.7 | 20.9 | 20.3 | -0.6 |
|  | 2008 | 21.2 | 34.8 | 13.6 | 21.4 | 22 | 0.6 |
|  | 2009 | 20.1 | 33.5 | 13.4 | 21.3 | 21.5 | 0.2 |
|  | 2010 | 20.9 | 33.8 | 12.9 | 21.9 | 21.1 | -0.8 |
|  | 2011 | 21 | 34.4 | 13.4 | 21.1 | 21.2 | 0.1 |
|  | 2012 | 21.2 | 34.4 | 13.2 | 22.2 | 22.5 | 0.3 |
| 30 mg/kg | 3001 | 21.7 | 30.4 | 8.7 | 21.7 | 21.4 | -0.3 |
|  | 3002 | 20.9 | 31 | 10.1 | 21.5 | 21.4 | -0.1 |
|  | 3003 | 21.2 | 30.1 | 8.9 | 22 | 20.8 | -1.2 |
|  | 3004 | 22 | 30.3 | 8.3 | 21.2 | 20.8 | -0.4 |
|  | 3005 | 20.6 | 34.9 | 14.3 | 21.6 | 21.3 | -0.3 |
|  | 3006 | 22.4 | 34.2 | 11.8 | 21.2 | 21.2 | 0 |
|  | 3007 | 21.3 | 30.4 | 9.1 | 21.7 | 21.7 | 0 |
|  | 3008 | 22.7 | 33.3 | 10.6 | 22 | 21.2 | -0.8 |
|  | 3009 | 20.6 | 33.7 | 13.1 | 21.4 | 20.8 | -0.6 |
|  | 3010 | 21.1 | 34.8 | 13.7 | 21.2 | 20.7 | -0.5 |
|  | 3011 | 21.4 | 33.2 | 11.8 | 21.9 | 20.7 | -1.2 |
|  | 3012 | 21.6 | 33.9 | 12.3 | 21.5 | 21.8 | 0.3 |
| 60 mg/kg | 4001 | 20.7 | 30.6 | 9.9 | 21.3 | 21.4 | 0.1 |
|  | 4002 | 21.4 | 30.4 | 9 | 21.4 | 21.3 | -0.1 |
|  | 4003 | 21.4 | 30.7 | 9.3 | 20.8 | 21.1 | 0.3 |
|  | 4004 | 21.7 | 30.6 | 8.9 | 21.2 | 21.9 | 0.7 |
|  | 4005 | 21.3 | 33.9 | 12.6 | 21.6 | 21 | -0.6 |
|  | 4006 | 21.4 | 34.9 | 13.5 | 21.2 | 21 | -0.2 |
|  | 4007 | 21.4 | 30.8 | 9.4 | 20.5 | 20.7 | 0.2 |
|  | 4008 | 20.4 | 30.4 | 10 | 21.1 | 21.5 | 0.4 |
|  | 4009 | 21.2 | 34.6 | 13.4 | 21.6 | 21 | -0.6 |
|  | 4010 | 21.2 | 33.9 | 12.7 | 20.6 | 20.4 | -0.2 |
|  | 4011 | 20.4 | 33.2 | 12.8 | 21.2 | 19.8 | -1.4 |
|  | 4012 | 20.4 | 32.9 | 12.5 | 21.6 | 20.9 | -0.7 |

**Supplementary Table 3: Δ IOP in Morrison model rats**

Δ IOP mmHg (OD-OS) per Animal

| Treatment | **Subject Name** | **Δ IOP3**  **mmHg (Day21)** | **Δ IOP4**  **mmHg (Day 29)** | **Δ IOP5**  **mmHg (Day35)** | **Δ IOP6**  **mmHg (Day41)** | **Average**  **ΔIOP (3-6)**  **mmHg** | **Eligible for Immunostaining** |
| --- | --- | --- | --- | --- | --- | --- | --- |
|  | 1001 | 11.0 | 13.1 | 12.1 | 13.2 | 12.4 | yes |
|  | 1002 | 15.3 | 10.4 | 13.2 | 12.3 | 12.8 | yes |
|  | 1003 | 12.7 | 12.2 | 12.4 | 13.3 | 12.7 | yes |
|  | 1004 | 11.6 | 10.9 | 12.1 | 12.9 | 11.9 | yes |
| DDW | 1005 | 12.8 | 10.5 | 11.7 | 12.5 | 11.9 | yes |
|  | 1006 | 8.6 | N/A | N/A | N/A | N/A | N/A |
|  | 1007 | 12.3 | 11.4 | 13.7 | 11.5 | 12.2 | yes |
|  | 1008 | 12.8 | 12.3 | 13.1 | 11.8 | 12.5 | yes |
|  | 1009 | 9.6 | 11.3 | 13.0 | 13.6 | 11.9 | yes |
|  | 1010 | 10.7 | 12.3 | 13.4 | 13.2 | 12.4 | yes |
|  | 1011 | 14.5 | 13.4 | 12.0 | 12.6 | 13.1 | yes |
|  | 1012 | 12.9 | 10.9 | 10.6 | 12.4 | 11.7 | yes |
|  | 2001 | 12.8 | 13.4 | 12.2 | 11.8 | 12.6 | yes |
|  | 2002 | 11.8 | 12.7 | 12.7 | 12.3 | 12.4 | yes |
|  | 2003 | 12.9 | 15.6 | 14.0 | 11.1 | 13.4 | yes |
| Pridopidine | 2004 | 13.7 | 11.1 | 10.7 | 13.8 | 12.3 | yes |
| 3 mg/kg | 2005 | N/A | N/A | N/A | N/A | N/A | N/A |
|  | 2006 | 12.4 | 12.0 | 12.7 | 12.0 | 12.3 | yes |
|  | 2007 | 10.7 | 13.6 | 12.3 | 10.2 | 11.7 | yes |
|  | 2008 | 12.8 | 9.5 | 12.7 | 12.6 | 11.9 | yes |
|  | 2009 | 11.2 | 12.1 | 12.9 | 12.6 | 12.2 | yes |
|  | 2010 | 14.1 | 12.7 | 11.2 | 13.4 | 12.9 | yes |
|  | 2011 | 12.8 | 13.4 | 13.3 | 12.7 | 13.1 | yes |
|  | 2012 | 13.0 | 10.8 | 13.9 | 12.4 | 12.5 | yes |
|  | 3001 | 12.6 | 12.5 | 12.8 | 12.2 | 12.5 | yes |
|  | 3002 | 13.1 | 11.8 | 10.0 | 13.5 | 12.1 | yes |
|  | 3003 | 13.5 | 13.5 | 11.5 | 11.1 | 12.4 | yes |
|  | 3004 | 11.9 | 13.6 | 12.5 | 13.0 | 12.8 | yes |
| Pridopidine | 3005 | 13.0 | 13.1 | 10.1 | 12.3 | 12.1 | yes |
| 30 mg/kg | 3006 | 9.5 | 13.6 | 12.7 | 12.5 | 12.1 | yes |
|  | 3007 | 13.6 | 12.4 | 11.5 | 12.6 | 12.5 | yes |
|  | 3008 | 12.4 | 12.5 | 12.3 | 12.2 | 12.4 | yes |
|  | 3009 | 14.5 | 13.1 | 12.0 | 12.2 | 13.0 | yes |
|  | 3010 | 10.9 | 11.2 | 10.8 | 14.0 | 11.7 | yes |
|  | 3011 | 11.1 | 15.1 | 12.1 | 10.9 | 12.3 | yes |
|  | 3012 | 13.8 | 11.6 | 11.7 | 12.6 | 12.4 | yes |
|  | 4001 | N/A | N/A | N/A | N/A | N/A | N/A |
|  | 4002 | 13.6 | 13.5 | 12.2 | 12.8 | 13.0 | yes |
|  | 4003 | 13.0 | 12.9 | 12.5 | 12.9 | 12.8 | yes |
|  | 4004 | 11.8 | 13.1 | 11.6 | 13.0 | 12.4 | yes |
| Pridopidine | 4005 | 11.0 | 12.5 | 11.9 | 10.5 | 11.5 | yes |
| 60 mg/kg | 4006 | 11.3 | 11.9 | 11.8 | 13.2 | 12.1 | yes |
|  | 4007 | 13.7 | 13.0 | 11.7 | 12.8 | 12.8 | yes |
|  | 4008 | 12.6 | 11.7 | 11.8 | 12.0 | 12.0 | yes |
|  | 4009 | 12.6 | 11.9 | 11.7 | 12.1 | 12.1 | yes |
|  | 4010 | 12.2 | 12.1 | 11.7 | 12.3 | 12.1 | yes |
|  | 4011 | 14.3 | 12.5 | 11.3 | 13.1 | 12.8 | yes |
|  | 4012 | 12.1 | 12.3 | 13.1 | 12.2 | 12.4 | yes |

**Supplementary Table 4: Summary of RGC counts per group in Morrison model**

| **Group** | **Control** | | **3 mg/kg** | | **30 mg/kg** | | **60 mg/kg** | |
| --- | --- | --- | --- | --- | --- | --- | --- | --- |
| **Eye** | **OD** | **OS** | **OD** | **OS** | **OD** | **OS** | **OD** | **OS** |
| **n rats** | 11 | | 8 | | 9 | | 10 | |
| **Total images analyzed** | 62 | 74 | 47 | 49 | 49 | 52 | 62 | 72 |
| **Mean RGC count/animal** | 82.82 | 146.82 | 105.25 | 144.75 | 122.78 | 158.56 | 116.50 | 130.40 |
| **SD** | 28.32 | 24.13 | 22.49 | 25.10 | 24.21 | 18.72 | 35.08 | 34.52 |

**Supplementary Table 5: Summary of body weights – LC model**

|  | |  | |  | |  |  |  |
| --- | --- | --- | --- | --- | --- | --- | --- | --- |
|  |  | |  | | Pridopidine | | | |
| Day |  | | Vehicle | | 3 mg/kg | | 30 mg/kg | 60 mg/kg |
| 0 | Mean (g) | | 520 | | 539 | | 527 | 535 |
|  | STDEV | | 39 | | 41 | | 36 | 28 |
| 7 | Mean (g) | | 543 | | 551 | | 539 | 537 |
|  | STDEV | | 39 | | 41 | | 33 | 26 |
| 14 | Mean (g) | | 546 | | 559 | | 538 | 536 |
|  | STDEV | | 41 | | 40 | | 34 | 27 |
